# Magnetic resonance imaging insights from active surveillance of women with ductal carcinoma in situ

**DOI:** 10.1038/s41523-024-00677-9

**Published:** 2024-08-04

**Authors:** Heather I. Greenwood, Cristian K. Maldonado Rodas, Rita I. Freimanis, Alexa C. Glencer, Phoebe N. Miller, Rita A. Mukhtar, Case Brabham, Christina Yau, Jennifer M. Rosenbluth, Gillian L. Hirst, Michael J. Campbell, Alexander Borowsky, Nola Hylton, Laura J. Esserman, Amrita Basu

**Affiliations:** 1 University of California San Francisco Department of Radiology, San Francisco, CA USA; 2 University of California San Francisco Department of Surgery, San Francisco, CA USA; 3 Harvard Medical School, Boston, MA USA; 4 University of California San Francisco Department of Medicine, San Francisco, CA USA; 5 University of California Davis Department of Pathology, Sacramento, CA USA

**Keywords:** Prognostic markers, Breast cancer

## Abstract

New approaches are needed to determine which ductal carcinoma in situ (DCIS) is at high risk for progression to invasive ductal carcinoma (IDC). We retrospectively studied DCIS patients who declined surgery (2002–2019), and received endocrine therapy (ET) and breast MRI. Baseline MRI and changes at 3 months and 6 months were analyzed by recursive partitioning to stratify IDC risk. Sixty-two patients (63 DCIS; 1 bilateral) with a mean follow-up of 8.5 years were included. Fifty-one percent remained on active surveillance (AS) without evidence of IDC, with a mean duration of 7.6 years. A decision tree based on MRI features of lesion distinctness and background parenchymal enhancement (BPE) at baseline and change after 3 months of ET stratified patients into low, intermediate, and high risk for progression to IDC. MRI imaging features in patients treated with ET and undergoing AS, may help determine which DCIS lesions are at low versus high risk for IDC.

## Introduction

Prior to the advent of screening mammography, ductal carcinoma in situ (DCIS) accounted for <5% of all breast cancers^1^. With the introduction and acceptance of widespread screening mammography, DCIS now accounts for 25–30% of screen-detected breast cancers in the United States^2^. DCIS is a non-invasive and non-obligate precursor to the development of invasive ductal carcinoma (IDC)^2,3^. Currently, it is not possible to predict which DCIS lesions will progress to invasive carcinoma and which will not^4^. Despite the varying risk of progression these lesions pose, the standard treatment of DCIS consists of local therapy similar to that for invasive cancer—with mastectomy or lumpectomy and radiation therapy. It is important to note that women with DCIS are at elevated risk for developing invasive breast cancer, even after breast conservation surgery^5^. Our inability to predict which lesions progress to invasive disease means that, in some women with DCIS, “standard therapy” likely constitutes overtreatment^6,7^.

The most common mammographic presentation of DCIS is microcalcifications; however, mammography is limited in detecting non-calcified lesions, especially in the setting of dense breasts. Breast magnetic resonance imaging (MRI) has been shown to be the most sensitive modality for the detection of DCIS, as it relies on contrast enhancement and is, therefore, not limited by dense breast tissue. Previously, MRI showed lower sensitivity of detection of DCIS compared to IDC (71%–87.8%)^8,9^, but sensitivities for DCIS detection have now improved with higher spatial resolution MRIs^10^. In addition, the majority of DCIS missed by MRI has been shown to be low-grade DCIS^11^. Not only is MRI the most sensitive imaging exam for the detection of DCIS, but it also provides other clinically relevant information, including background enhancement and the distribution of non-mass and mass enhancement bilaterally^12^. Contrast enhancement is a biomarker of angiogenic and protease activity, and protease activity is required for cancerous cells to penetrate the basement membrane and invade beyond it. DCIS lesions that enhance and are mass-like on MRI may thus have proteolytic and/or vessel recruitment properties that make them more likely to progress to invasive cancer than those detected by mammography alone^13^.

Ultrafast MRI (UF-MRI) is a new MRI technique that also relies on contrast enhancement and, thus tumor angiogenesis. Recent studies have shown promising applications of this technique to predict the upgrade of pure DCIS to invasive disease at surgery. Time to enhancement (TTE) in UF-MRI describes the time from aortic enhancement to first lesion enhancement. Shorter TTE of DCIS lesion on pre-operative UF-MRI can predict invasive disease at surgery, with a threshold of 11 seconds providing maximum specificity (50%) and sensitivity (76%) for upgrade - a promising imaging biomarker^14^.

Another important controversy in DCIS is the diagnostic criteria that separate atypical ductal hyperplasia, which some consider a global risk factor for breast cancer, and low-grade DCIS, considered a focal indicator of breast cancer risk^15^. Given that MRI is the most sensitive imaging modality to detect invasive cancer (and to determine background enhancement), it may be a useful tool for evaluating and advising patients as to whether they have underlying invasive disease, focal disease, and/or diffuse enhancement. MRI surveillance can provide a platform to introduce neoadjuvant treatment as an initial therapy for DCIS, to determine the benefit of surgical intervention and to identify candidates who can reasonably be offered active surveillance (AS) of their DCIS with serial imaging.

These efforts can be improved by incorporating an assessment of background parenchymal enhancement (BPE) on breast MRI, which is a physiologic phenomenon in which normal breast tissue demonstrates a signal related to the uptake of contrast as part of the AS platform. BPE is routinely reported on all clinical breast MRIs with standardized BI-RADS (Breast Imaging Reporting and Data System) categories of minimal, mild, moderate, or marked^16^. BPE is affected by both endogenous and exogenous hormone level changes^17^. Studies have shown that elevated BPE is associated with increased breast cancer risk^18,19^. Given that BPE is routinely measured on MRI and is a modifiable measure of risk, it may be a good candidate for an early endpoint to measure the effectiveness of breast cancer risk-reducing agents such as endocrine therapy (ET).

At our institution, we have a large database of patients enrolled in imaging studies for DCIS, including women who have declined surgical resection at diagnosis and instead accepted being placed on standard endocrine treatment (ET) and were subsequently assigned a protocol of AS with intensive serial imaging, including breast MRI. The purpose of our study was, therefore, to utilize this diverse population of patients with DCIS to identify features on breast MRI that may help to distinguish DCIS lesions with low versus high risk of progressing to IDC in women on ET. We have previously reported that the presence of a mass is a risk factor for the presence of or risk for invasive cancer^20^. The clinician leading the study observed that both patterns of BPE and lesion varied over time and that many patients did not present with a mass on MRI and suggested that both lesion conspicuity and BPE at baseline and over the course of time, together, could provide insight to risk over time. Patients in our cohort went to the operating room for various reasons: (1) Concern about the progression of disease on imaging; or (2) patient anxiety about remaining on AS. The goal of this study was to record baseline as well as changes in BPE and characterize the initial lesion at baseline and changes over time in patients on ET undergoing AS and determine if these patterns could provide insight into risk assessment. We tested the hypothesis that initial patterns, as well as change or lack of change on MRI in the lesion and the BPE, correlate with the risk of progression of DCIS lesions.

## Results

### Patient population and lesion characteristics

Of the 71 (73 lesions as there were two bilateral DCIS) patients in the UCSF AS population, 62 patients (63 lesions; 1 case of bilateral DCIS) met the predefined inclusion criteria of receiving ET and having at least 2 sequential MRI scans available (Supplementary Fig. 1). All nine patients excluded from this analysis did not receive ET.

Patient and DCIS characteristics for those included in our cohort are listed in Table 1. Of note, the mean age of DCIS diagnosis was 53.8 years (range 29.8–78.8 years), the mean follow-up time from the date of diagnosis was 8.5 years (range 2.1–21.5 years), and the meantime on AS was 4.9 years (range of 0.2-19.3 years). All 62 patients (100%) were on ET for at least 3 years (range 3–5 years). Thirty-three patients (33/63 cases, 52.4%) were on estrogen modulators, including tamoxifen (*N* = 28) and raloxifene (*N* = 5). Twenty-nine patients (29/63 cases, 46.0%) were on aromatase inhibitors, including letrozole (*N* = 23), anastrozole (*N* = 4), and exemestane (*N* = 2). One patient (1/63 cases, 1.5%) was on an unknown type of ET. Sixty (96.7%) patients (61 lesions, 96.8%) had hormone receptor (HR) positive DCIS, and two patients (3.2%) had DCIS with unknown HR status. One of the patients who had DCIS with unknown HR status had low-grade DCIS, while the other patient had intermediate-grade DCIS and thus was assumed to be endocrine positive. Twenty-seven (43.5%) patients (1 bilateral case) were premenopausal, and 35 (56.5%) were postmenopausal at the time of diagnosis. Thirty-one (49.2%) cases of DCIS went to surgery, and 32 (50.8%) cases of DCIS are still being monitored on AS. Of the 31 cases of DCIS who went to surgery, half, or 16 (51.6%) cases, had IDC, and 15 (48.4%) cases had DCIS on their final surgical pathology report. The mean time to surgery was 2.2 years (range 0.2–6.7 years). All 32 cases of DCIS that did not have surgery are still alive, on AS, and being evaluated with serial imaging.

**Table 1:** Demographic and clinical characteristics for active surveillance cohort at the time of diagnosis

Data category	Cohort (*n* = 63)
Mean age at diagnosis (range in years)	53.8 (29.8–78.8)
Age <50	25 (39.7%)
Age ≥50	38 (60.3%)
Mean follow–up (range in years)	8.5 (2.1–21.5)
Mean time on AS total (range in years)	4.9 (0.2–19.3)
Mean time on AS before surgery (range in years)	2.2 (0.2–6.7)
Mean time on AS and no surgery (range in years)	7.6 (2.1–19.3)
Menopausal status	
Premenopausal	28 (44.4%)
Postmenopausal	35 (55.6%)
HR status	
Positive	61 (96.8%)
Negative	0 (0.0%)
Unknown	2 (3.2%)
HER2 status	
Positive	7 (11.1%)
Negative	22 (34.9%)
Unknown*	34 (54.0%)
Grade	
High	18 (28.6%)
Intermediate	32 (50.8%)
Low	11 (17.4%)
Unknown	2 (3.2%)
Surgery	
Yes	31 (49.2%)
No	32 (50.8%)

### MRI features and risk groups

**Fig. 1:**
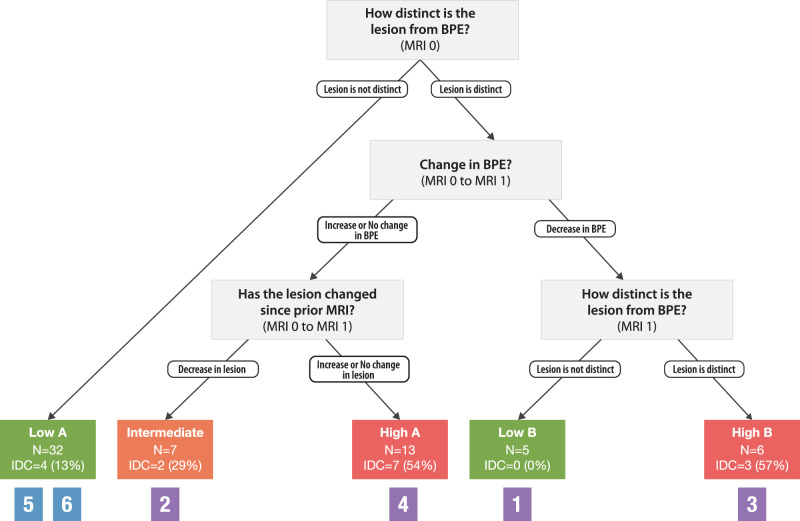
Decision tree (*N* = 62 patients; 63 lesions) adapted from a larger cohort (*N* = 71 patients; 73 lesions) using a recursive partitioning (R-PART) decision tree that uses baseline and post-treatment imaging features. Reader answers to the MRI questions assessed at baseline (MRI 0) and post-treatment (MRI 1) that classified risk buckets based on the likelihood of progression to invasive cancer. Questions considering MRI features, including the following: change in BPE, lesion distinctness, and change in lesion size, were considered. Groups Low A, High A, Low B, High B, and High C. Colored boxes under risk buckets represent clinical interpretations after endocrine exposure (Fig. 2B).

To better understand the performance of the rules established in our previous study^21^, within an endocrine-treated sub-population, we constructed a tree using 4 MRI features (lesion distinctness at time points at baseline and 3 months, change in BPE between baseline and 3 months, and change in lesion between baseline and 3 months) which resulted in patients being stratified into five risk groups (Low A, Low B, Intermediate, High A, and High B) as seen in Fig. 1. Groups deemed as lower risk had less than or equal to 12.5% of cases progress to IDC, groups deemed as intermediate had a 28% risk of progressing to IDC, and groups deemed as higher risk had an average of 52% chance of cases progressing to IDC. Group Low A (*N* = 32) identified patients without a distinct lesion on MRI. Group Low B (*N* = 5) included patients who had a distinct lesion at baseline, and a decrease in BPE resolution of the distinct lesion upon endocrine treatment. The Intermediate (*N* = 7) group included patients with a distinct lesion at baseline, no change or an increase in BPE, and a decrease in lesion size upon endocrine treatment. Group High A (*N* = 6) included patients who had a distinct lesion at baseline, a decrease in BPE, and a distinct lesion upon endocrine treatment. Group High B (*N* = 13) was enriched for patients with a distinct lesion at baseline, no change or an increase in BPE, combined with an increase in lesion size after endocrine treatment. The hypotheses that are referenced in Glencer et al.^21^ are shown in Supplementary Fig. 2. The grey boxes in Fig. 1 under each risk group are numbered on the basis of our conclusions in Supplementary Fig. 2.

**Fig. 2:**
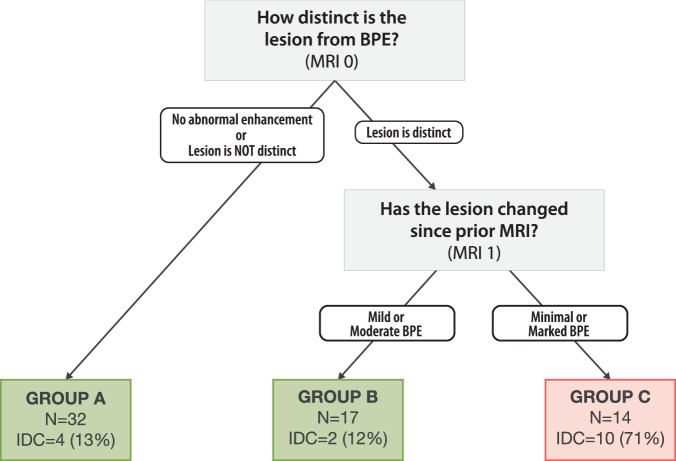
Patient classification from a recursive partitioning (R-PART) decision tree that uses baseline imaging features. Reader answers to the MRI questions assessed at baseline (MR0) were inputted into an R-PART algorithm that classified risk buckets based on the likelihood of progression to invasive cancer. Questions considering MRI features, including the following: background parenchymal enhancement (BPE), lesion distinctness, and likelihood of invasive cancer, were considered. The algorithm indicated that BPE and lesion distinctness were important baseline imaging features, and classified cases of DCIS into groups A, B, and C.

To evaluate whether baseline MRI features alone could risk stratifying our population, we performed recursive partitioning using only baseline variables as input (distinct lesion, quantitative BPE, BPE symmetry, likelihood of baseline invasive cancer). The performance of the model was assessed through root node error [percent of correctly sorted records at the first (root) splitting node] (error = 0.15) multiplied by the cross-validation error (average error = 1.0), a predictive measure of accuracy. The resulting classification tree includes 2 variables and classifies patients into 3 groups (Fig. 2). Three different categories (Group A, B, and C) were created using these MRI features. In Group A, there were no lesions that were distinct above BPE. All cases in Group B had lesions that were distinct above BPE. Both Group A (*N* = 32) and Group B (*N* = 17) had low rates of invasive cancers (14.5% and 11.8% respectively). Combining Group A and Group B, there were a total of 6 patients (12.2%, 6/49) diagnosed with invasive cancer over the course of the study. In contrast, the presence of invasive cancer was much higher in Group C (10/14, 71.4%). All lesions in Group C were distinct above marked BPE or were distinct above minimal BPE and did not shrink in response to endocrine risk-reducing medication. The combination of minimal BPE and a distinct lesion above BPE was enriched (11/14, 78.6%) in Group C. Invasive cancer characteristics for each group can be seen in Supplementary Table 3.

**Fig. 3:**
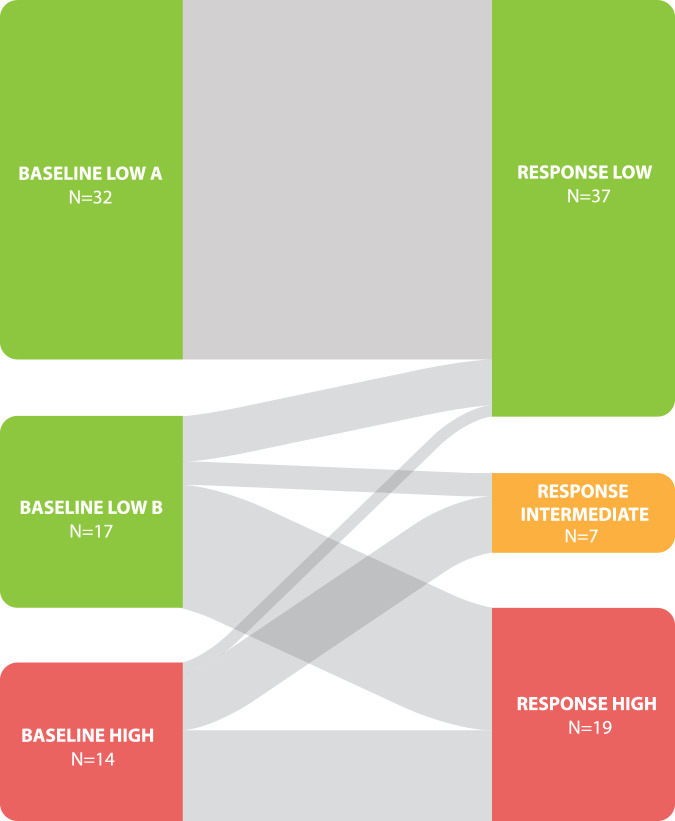
Patient re-classification of risk after endocrine exposure. Cases of DCIS were initially classified into groups A, B, and C from the baseline decision tree in Fig. 2B and were then re-classified into groups Low A, Low B, Intermediate, High A, and High B based on their response to endocrine therapy. Low–post-treatment is composed of Low A and Low B, Intermediate–Post-treatment is composed of Intermediate only, and high–post-treatment is composed of High A and High B. The Width of the flow is proportional to the quantity presented.

We next analyzed whether the patients who were characterized as high risk at baseline remained as high risk, or whether exposure to ET could modify the initial predicted risk. DCIS cases that were initially classified as lower risk (Groups A and B) and higher risk (Group C) by our baseline decision tree were re-classified into the following different risk groups post-treatment (Fig. 3). Of the cases that were initially categorized as high risk (Group C, *N* = 14) with baseline features only, 8 were distributed to intermediate risk group (*N* = 8/14, 57.1%), 1 moved to a low-risk group (*N* = 1/14, 7.1%), whereas 5 (*N* = 5/14, 35.7%) remained in a high-risk group (High B and High C). The evidence of DCIS patients moving between high-risk, intermediate, and low-risk groups (and vice versa) after ET shows that it can be worthwhile to offer ET to patients to determine if their overall risk decreases, increases, or does not change.

### Background parenchymal enhancements

**Fig. 4:**
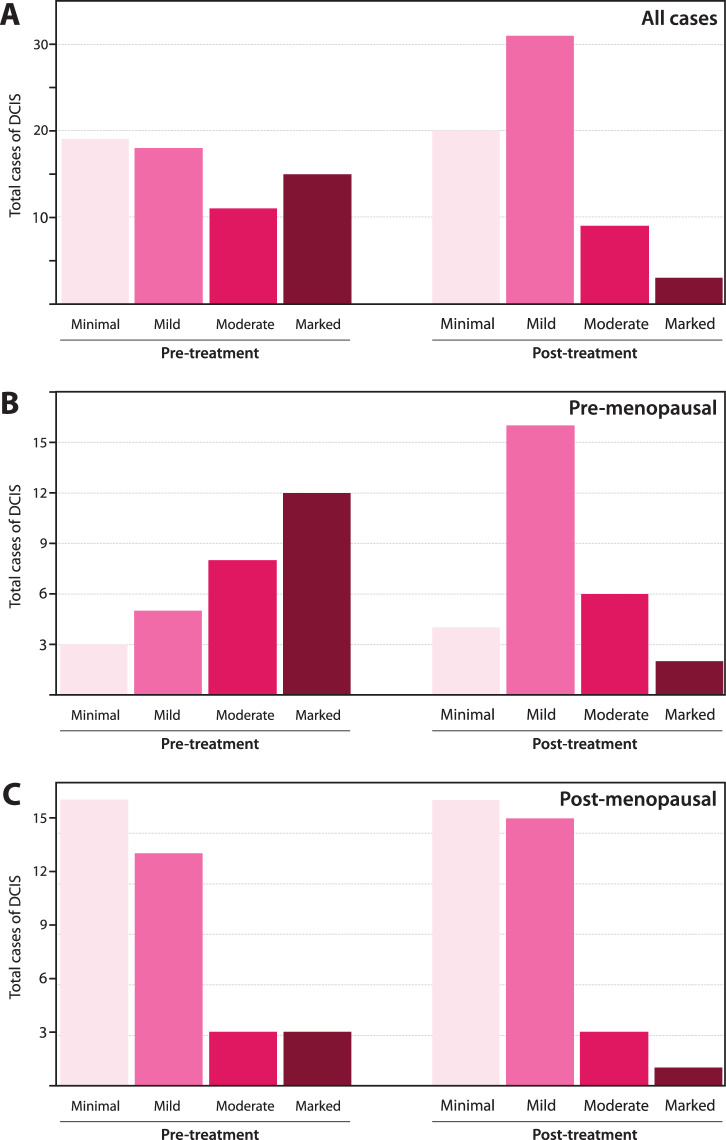
Background parenchymal enhancement changes after endocrine therapy. Bar distribution plot depicting the change in background parenchymal enhancement (BPE) categories before and after endocrine therapy exposure separated by (**A**) all cases of DCIS, (**B**) pre-menopausal cases of DCIS (*N* = 28), and (**C**) post-menopausal cases of DCIS (*N* = 35).

**Fig. 5:**
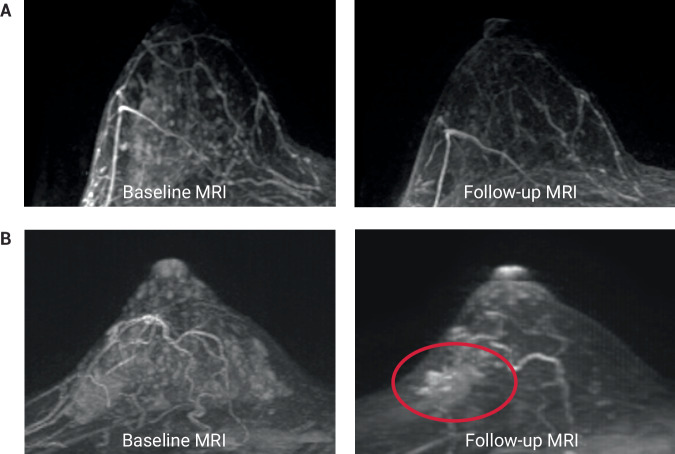
Changes in ipsilateral breast MRI features. Axial subtracted post-contrasts maximum intensity projection (MIP) images of the ipsilateral breast before and after endocrine therapy. One example shows marked BPE at baseline that is reduced after endocrine therapy with no distinct lesion prior to or following ET (**A**), and the other shows marked BPE with no distinct lesion above BPE at baseline and a reduction in BPE after endocrine therapy and a distinct lesion on follow-up (oval) (**B**).

Given that BPE is known to be associated with an increased risk for invasive breast cancer and is a modifiable measure of risk, we evaluated how BPE changed from baseline (MR0), to timepoint 1 (MR1), after endocrine exposure for 3 months. The changes in BPE after endocrine exposure can be seen in Fig. 4A for all cases of DCIS (*n* = 63), Fig. 4B for pre-menopausal cases of DCIS (*n* = 28), and Fig. 4C for post-menopausal cases of DCIS (*n* = 35). In Fig. 4A, 94.7% (18/19) of DCIS cases with minimal BPE and 77.8% (14/18) of DCIS cases with mild BPE stayed within that same BPE classification after endocrine exposure. However, 54.5% (6/11) of DCIS cases that had moderate BPE at baseline had mild BPE after endocrine exposure. Similarly, 66.7% (10/15) and 20% (3/15) of DCIS cases that had marked BPE at baseline had mild and moderate BPE after endocrine exposure, respectively. In the pre-menopausal group, we saw a higher proportion of cases with moderate (8/28, 28.6%) or marked (12/28, 42.6%) BPE at baseline in comparison to the post-menopausal group (3/35 or 8.6% with moderate BPE and 3/35 or 8.6% with marked BPE). In the post-menopausal group, we saw a higher proportion of cases with minimal (16/35, 45.7%) or mild (13/35, 37.1%) BPE at baseline compared to the pre-menopausal group (3/28 or 10.7% for minimal BPE and 5/28 or 17.6% for mild BPE). Patients with mild, moderate, or marked BPE at baseline had a reduction in BPE after 3-months of ET, likely reflective of the ability of ET to reduce a patient’s overall risk. Examples of BPE reduction in patients on AS with ET can be seen in Fig. 5.

### Inter-reader variability

We evaluated reader variability for the evaluated MRI features in this retrospective analysis. Scatter plots displaying the raw scoring of each reader (x- and y-axis) for the first three time points (MR0, MR1, and MR2) (Supplementary Fig. 3). On each scatter plot, we compare reader 1 (x-axis) and reader 2 (y-axis) scores for each patient, MRI feature and timepoint. By timepoint 2 (MR2), our readers had a percent agreement greater than 60% for all MRI features. BPE Symmetry (87.5%), new or progressed DCIS (87.23%), the likelihood of invasive cancer (91.49%), change in contralateral BPE (77.50%) and change in the lesion (78.95%) had the higher reader agreement values. Ipsilateral BPE (68.06%), contralateral BPE (65.00%), change in ipsilateral BPE (61.70%), and lesion distinctness (63.83%) had lower percent agreement values. Future confirmatory studies will include an evaluation of imaging features (e.g., ipsilateral BPE, lesion distinctness, change in a lesion, and change in ipsilateral BPE) to be assessed through semiquantitative endpoints.

## Discussion

Since the advent of screening mammography, the diagnosis and removal of thousands of DCIS lesions annually has not been accompanied by a decrease in invasive cancer incidence. This presents us with the opportunity to characterize and identify DCIS lesions that are of lower risk and amenable to treatment strategies, including AS with risk-reducing therapies and DCIS lesions that are of higher risk and better treated with surgical excision. Our study shows that MRI features after short-term exposure to ET may help to identify those DCIS lesions that are at high, low, or intermediate risk of progressing to invasive disease.

We found that MR imaging features in patients undergoing AS with ET may be used to assess whether DCIS lesions are amenable to AS. Lesions that are persistently focal (with “distinct from BPE” as a surrogate descriptor), or become more focal as the surrounding BPE resolves are at higher risk of progressing to invasive cancer, or having invasive cancer. Given that a standard approach to stage 2 and 3 endocrine-positive invasive breast cancer is the use of 6 months of neoadjuvant ET^22^, this is a safe approach and generates important information for patients and clinicians.

When looking at baseline imaging features, the presence of a distinct lesion in the setting of minimal or marked BPE placed patients into a group with a high subsequent risk of invasive cancer. Several studies in the literature have previously demonstrated that the presence of a mass at MRI in patients with diagnosed DCIS has been associated with invasive cancer^23–26^, which is consistent with our results showing a distinct lesion, which can be a mass, was a risk factor for invasive cancer. However, patients with a distinct lesion that either resolves or NME that reduces in response to ET are at lower risk for developing invasive cancer. Patients with no abnormal enhancement or no distinct lesion were at low risk for subsequent invasive cancer. A substantial fraction of patients fall into this category, and one of the benefits of MRI in the setting of DCIS may be to identify these patients who may be better served with endocrine risk reduction than with surgery. This seems intuitive as their lesion did not enhance, suggesting either lower invasive potential, lack of distinct features separating the lesion from normal tissue, or that the DCIS was largely removed at core biopsy. We note that there are some patients who had a distinct lesion and mild or moderate BPE at baseline that were assigned to lower-risk categories. These patients get reassigned to AS when follow-up images and responses to ET are taken into account.

Interestingly, when BPE reduced over time on ET without the presence of a distinct lesion (mass or NME) above the background or with a distinct lesion that also reduced over time, these findings predicted a low risk for the development of invasive cancer. A mass at baseline was found to be more predictive of cancer arising when patients were included who did not take ET. However, when BPE decreased on ET, and a distinct lesion persisted or developed, there was a higher risk for the development of an invasive tumor. This suggests that while the BPE is responding to ET, the lesion is not, and surgical therapy may be indicated due to intrinsic mechanisms of resistance within the DCIS itself.

From previous studies, we know that ET, especially tamoxifen, is known to suppress BPE. As BPE is reduced, the likelihood of agreement on categories of BPE and identification of a mass increases. Thus, it is not surprising that concordance increases with time on ET. Importantly, these imaging features can serve as early endpoints for efficacy, by measuring the change in BPE and the appearance of a distinct lesion. This can not only serve to assess impact of known endocrine risk reducing medications but can accelerate the testing of novel endocrine agents such as SERDs (selective endocrine receptor degraders), and androgen agonists and antagonists. We plan to conduct a study evaluating multiple endocrine agents in a multicenter study, using imaging agents as a way to contribute to the evaluation (the DCIS Re-Evaluation of Conditions for AS Suitability as Treatment [RECAST] study)^27^. As part of this study, we will have the opportunity to explore the biologic basis for the persistence of a distinct lesion in the setting of ET or for the failure of BPE to be reduced. Our results suggest that MRI features at baseline and over a short course of ET, based on changes in lesions and BPE, allow knowledge to be accrued that helps better assess which lesions should be treated with surgical management versus those that can be managed with a more global risk reduction approach using ET. Previous studies have shown the potential of using deep learning models with MRI to differentiate between pure DCIS and DCIS upgraded at surgery to invasive disease^28^. Based on the results of the RECAST study, future directions could include developing a deep learning model and incorporating imaging features of lesion distinctness, BPE, and change in both over time to help predict the risk of invasive disease.

These data also provide insight into which MRI features are the most predictive. This gives us the ability to refine the way in which BPE and lesion conspicuity are measured, as seen in Supplementary Table 4. In our study, it was anticipated that lesions would be largely NME, as most patients with a mass lesion would have been recommended for surgery over AS, given the higher association with underlying invasive disease. In the initial survey, that issue was addressed by asking, “What is the likelihood that there is invasive disease?” The finding of a mass would have led to a response of ‘high likelihood.’ Because there were so few masses in our study cohort, this question was not initially included, as it did not affect the outcome tree. But in follow-up studies, if a mass developed, that was noted and included in the analysis. From the concordance data, we learned that the assessment of these features improved with exposure to ET, which made the lesion features easier to assess.

Frequent MRIs were performed on our patients undergoing AS, as this was a new approach to managing DCIS. Most commonly patients underwent a baseline MRI at diagnosis, MRI at 3 months, 6 months, and then MRIs were more spread out to every 6-12 months. There was some variability in the imaging frequency between patients. To minimize the risk of missing progression, imaging was performed frequently. Patients were not charged clinically, as this was performed under a research protocol. The purpose of analyzing the MR features was to come up with a safe way to follow patients on AS and to generate an imaging protocol that is practical. We found that the information was largely generated by MRI changes at the initial 3 and 6-month scans. We do note that for some patients, there may be barriers to the frequent use of MRI, including MRI tolerance (iv contrast, claustrophobia) and cost.

Our study has several limitations. First, it was a retrospective study at a single institution with only 2 readers. We have designed a prospective assessment in the context of a multi-institution study and will include the refined MRI features that will be semiquantitative and likely to be more reproducible. This was a non-randomized study, that included patients who sought to be on AS. In addition, readers were blinded to ultrasound and mammogram findings, which is not the norm in clinical practice, going forward readers should have access to these imaging modalities and all imaging should be interpreted together as is standard of practice in routine clinical care.

Future work will include multi-institution prospective trials to help confirm and determine MR imaging features that may aid in appropriately identifying which DCIS lesions need surgical treatment versus which may be safe to remain on imaging surveillance. These should include both qualitative and quantitative data. In addition, we hope to evaluate new emerging endocrine therapies that may be more tolerable to patients.

In conclusion, our study suggests that MR imaging features, specifically lesion distinctness at baseline and change over time in the context of BPE, in patients being treated with ET and undergoing AS, may help determine which DCIS lesions are at high risk for the development of invasive disease and are better managed with surgery versus lesions which may represent a global risk and are better managed with global risk reduction (ET). This is an important contribution to efforts to better personalize therapy for women diagnosed with DCIS and avoid overtreatment.

## Methods

### Cohort selection

This was an IRB-approved retrospective study of patients with biopsy-proven DCIS who chose not to undergo surgical treatment and instead enrolled in imaging surveillance studies at the University of California San Francisco between 2002 and 2019. A total of 188 women (190 cases of DCIS, 2 women had bilateral DCIS) enrolled in and received an MRI under one of 5 imaging surveillance study protocols. All patients provided written consent for their clinical, imaging, and pathological data to be used for research purposes. Patients underwent frequent clinical evaluations, including in-person consultations and physical exams, and serial breast MRI every three months, which started with a physical examination and MRI within 7 months of the initial clinic consultation. Other imaging, such as mammography or ultrasound, which may have also been performed for routine clinical purposes, is not included in this evaluation as the utility of MRI was the focus of the present study. All patients were offered endocrine risk-reducing therapy during their course of treatment.

The results of a cohort of 71 women (73 cases of DCIS, as 2 women had bilateral DCIS) who enrolled in AS imaging studies at UCSF have been previously reported^21^. In this cohort of 62 women (63 cases of DCIS: 1 bilateral case), we included only the subset of patients who received ET (from the 71 women cohort), had 2 or more breast MRI scans, and had at least two years of follow-up imaging from the time of diagnosis. Demographic information, pathological reports, imaging reports, and clinic evaluations were obtained from patient medical records. The primary outcome was the identification of invasive breast cancer.

### Study oversight

This study was approved by the Institutional Review Board of the UCSF Human Research Protection Program. It complies with all local and national regulations regarding the use of human study participants and was conducted in accordance with the criteria set by the Declaration of Helsinki. The authors of this manuscript vouch for the accuracy and completeness of the data reported. All patients provided written consent for their clinical, imaging, and pathological data to be used for research purposes.

### Imaging

Breast MRI scans were all performed on either 1.5 T or 3.0 T magnets. Each scan included standard breast MRI sequences, including fat-suppressed T2-, non-fat suppressed T1-, fat-suppressed pre-contrast T1-, and fat-suppressed T1-weighted post-contrast images with at least two post-contrast time points.

All women had at least 2 MRIs following DCIS diagnosis (without surgical intervention) (74.6% had 3 or more). If the patient had more than two MRIs, the breast radiologists interpreted a maximum of 5 MRIs for each patient. For women with more than 5 MRIs, the first 4 MRIs and the last one before surgery or the last one recorded while they were still on AS were used. This decision was made assuming that the last MRI prior to surgery would have findings that led to a change in treatment (i.e., surgical management). If the patient did not go to surgery, the most recent MRI while on AS was analyzed.

Two radiologists (HG, RF), sub-specialized in breast imaging with 8—31 years of experience, retrospectively analyzed all breast MRIs and were blinded to clinical outcomes. MRI features, including lesion conspicuity, BPE, change in lesion between MRIs, change in BPE between MRIs, and likelihood of invasive cancer, were subjectively measured independently by each breast radiologist. The exact questions posed are included in Supplementary Table 1. For lesion distinctness, radiologists looked for abnormal enhancement on MRI that they thought was associated with the surveilled DCIS lesion. In addition, for BPE, the radiologists subjectively assessed BPE for both contralateral and ipsilateral breasts per the standardized BI-RADS categories of minimal, mild, moderate, and marked. Of note, radiologists were provided the laterality of the DCIS but were blinded to other imaging modalities (mammogram and ultrasound).

To measure concordance between radiologist reads, we assessed the first three time points for each patient, where both radiologists independently evaluated each MRI feature. To compute a percent agreement between the two radiologists, we evaluated questions where radiologists were in exact agreement (radiologist A scored 3 and radiologist B scored 3), divided by the total number of patients being evaluated at that time point. Responses given by reader 1 (x-axis) and reader 2 (y-axis) were plotted via scatter plot. In addition, as seen in Supplementary Table 2, proportionality tests were conducted to evaluate whether IDC was associated with grade, age, and breast composition.

### Statistical analysis

R-PART is a statistical tool to provide rank ordering of the importance of biologic features in the model that predict a prespecified outcome. It is a decision tree analysis tool used to classify a population into homogenous subpopulations according to the association between a set of variables used for prediction and a dependent variable (outcome). The R-PART tool reveals the most predictive features, and how the variables relate to each other (conditional dependence). The result is a grouping of the variables, used in a particular order, that best predicts a given outcome variable. These partitions are done recursively until a form of the decision tree with the desired fit is reached. The optimal partitions are chosen from all possible partition options^29^.

We ran R-PART with baseline features and with information from timepoints 0, 3, and 6 months^21^. We also ran R-PART with baseline features alone, which included all MRI features at the first time point (pre-therapy). This included BPE for contralateral and ipsilateral breast, how distinct the lesion is from BPE, and the likelihood of baseline invasive cancer on MRI. We also included biological features, including grade, ER status, PR status, HER2 status, and menopausal status, of the DCIS. These features were not prioritized by the R-PART algorithm. The performance of the model was assessed through root node error [percent of correctly sorted records at the first (root) splitting node] multiplied by the cross-validation error, a predictive measure of accuracy.

To better understand performance of the rules established in our previous study^21^ within an endocrine-treated sub-population, we included in the list of dependent variables, a set of MRI features at baseline and 3 months as well as changes to the lesion (if present) relative to changes in BPE. This allowed the model to incorporate new evaluation points that were based on change over time with ET by comparing a previous time point. The features were a change in BPE, a change in the lesion, and a distinct lesion at baseline and at 3 months.

### Supplementary information

Online supplement

## Data Availability

Datasets used and/or analysed for the current study are available from the corresponding author upon reasonable request.

## Code availability

Analytic code used in the current study is available from the corresponding author upon reasonable request.

## References

[1] Swallow, C. J., Zee, K. J. V., Sacchini, V. & Borgen, P. I. Ductal carcinoma in situ of the breast: progress and controversy. *Curr. Prob. Surg.* **33**, 555–600 (1996). 10.1016/S0011-3840(05)80019-X 8765465

[2] Virnig, B. A., Tuttle, T. M., Shamliyan, T. & Kane, R. L. Ductal carcinoma in situ of the breast: a systematic review of incidence, treatment, and outcomes. *J. Natl. Cancer Inst.* **102**, 170–178 (2010). 20071685 10.1093/jnci/djp482

[3] Leonard, G. D. & Swain, S. M. Ductal carcinoma in situ, complexities and challenges. *J. Natl. Cancer Inst.* **96**, 906–920 (2004). 15199110 10.1093/jnci/djh164

[4] Nakhlis, F. & Morrow, M. Ductal carcinoma in situ. *Surg. Clin. N. Am.* **83**, 821–839 (2003). 12875598 10.1016/S0039-6109(03)00072-0

[5] Ernster, V. L., Barclay, J., Kerlikowske, K., Wilkie, H. & Ballard-Barbash, R. Mortality among women with ductal carcinoma in situ of the breast in the population-based surveillance, epidemiology and end results program. *Arch. Intern. Med.* **160**, 953–958 (2000). 10761960 10.1001/archinte.160.7.953

[6] Hamdy, F. C. et al. 10-year outcomes after monitoring, surgery, or radiotherapy for localized prostate cancer. *N. Engl. J. Med.* **375**, 1415–1424 (2016). 27626136 10.1056/NEJMoa1606220

[7] Ozanne, E. M. et al. Characterizing the impact of 25 years of DCIS treatment. *Breast Cancer Res. Treat.* **129**, 165–173 (2011). 21390494 10.1007/s10549-011-1430-5

[8] Obdeijn, I.-M. A. et al. Assessment of false-negative cases of breast MR imaging in women with a familial or genetic predisposition. *Breast Cancer Res. Treat.* **119**, 399 (2009). 10.1007/s10549-009-0607-7 19876732

[9] Wurdinger, S., Kamprath, S., Eschrich, D., Schneider, A. & Kaiser, W. A. False-negative findings of malignant breast lesions on preoperative magnetic resonance mammography. *Breast* **10**, 131–139 (2001). 14965573 10.1054/brst.2000.0232

[10] Lehman, C. D. Magnetic resonance imaging in the evaluation of ductal carcinoma in situ. *JNCI Monogr.* **2010**, 150–151 (2010). 10.1093/jncimonographs/lgq030 PMC5161071 20956821

[11] Kuhl, C. K. et al. MRI for diagnosis of pure ductal carcinoma in situ: a prospective observational study. *Lancet* **370**, 485–492 (2007). 17693177 10.1016/S0140-6736(07)61232-X

[12] Esserman, L. J. et al. Magnetic resonance imaging captures the biology of ductal carcinoma in situ. *J. Clin. Oncol.* **24**, 4603–4610 (2006). 17008702 10.1200/JCO.2005.04.5518 PMC4087112

[13] Kuhl, C. K. Why do purely intraductal cancers enhance on breast MR images? *Radiology* **253**, 281–283 (2009). 19864520 10.1148/radiol.2532091401

[14] Miceli, R., Gao, Y., Qian, K. & Heller, S. L. Predicting upgrade of ductal carcinoma in situ to invasive breast cancer at surgery with ultrafast imaging. *Am. J. Roentgenol.* **221**, 34–43 (2023). 36752370 10.2214/AJR.22.28698

[15] Khoury, T. Preneoplastic low-risk mammary ductal lesions (atypical ductal hyperplasia and ductal carcinoma in situ spectrum): current status and future directions. *Cancers* **14**, 507 (2022). 35158775 10.3390/cancers14030507 PMC8833401

[16] D’Orsi, C., Bassett, L., Feig, S. & others. Breast imaging reporting and data system (BI-RADS). *Breast imaging atlas*, 4th edn. American College of Radiology, Reston (2018).

[17] Liao, G. J. et al. Background parenchymal enhancement on breast MRI: a comprehensive review. *J. Magn. Reson. Imaging* **51**, 43–61 (2020). 31004391 10.1002/jmri.26762 PMC7207072

[18] Dontchos, B. N. et al. Are qualitative assessments of background parenchymal enhancement, amount of fibroglandular tissue on MR images, and mammographic density associated with breast cancer risk? *Radiology* **276**, 371–380 (2015). 25965809 10.1148/radiol.2015142304 PMC4554209

[19] Arasu, V. A. et al. Population-based assessment of the association between magnetic resonance imaging background parenchymal enhancement and future primary breast. *Cancer Risk. J. Clin. Oncol.* **37**, 954–963 (2019). 30625040 10.1200/JCO.18.00378 PMC6494266

[20] Kim, P. et al. Abstract P6-16-06: Improved early stratification of invasive cancer risk using MRI in a ductal carcinoma in-situ short-term active surveillance cohort treated with neoadjuvant endocrine therapy. *Cancer Res.* **80**, P6-16-06–P6-16–06 (2020). 10.1158/1538-7445.SABCS19-P6-16-06

[21] Glencer, A. C. et al. Identifying good candidates for active surveillance of ductal carcinoma in situ: insights from a large neoadjuvant endocrine therapy cohort. *Cancer Res. Commun.* **2**, 1579–1589 (2022). 36970720 10.1158/2767-9764.CRC-22-0263 PMC10035518

[22] Spring, L. M. et al. Neoadjuvant endocrine therapy for estrogen receptor–positive breast cancer: a systematic review and meta-analysis. *JAMA Oncol.* **2**, 1477 (2016). 27367583 10.1001/jamaoncol.2016.1897 PMC5738656

[23] Huang, Y.-T. et al. MRI findings of cancers preoperatively diagnosed as pure DCIS at core needle biopsy. *Acta Radiol.* **52**, 1064–1068 (2011). 21969708 10.1258/ar.2011.110213

[24] Wisner, D. J. et al. Features of occult invasion in biopsy‐proven DCIS at breast MRI. *Breast J.* **19**, 650–658 (2013). 24165314 10.1111/tbj.12201 PMC4036640

[25] Lee, C.-W. et al. Preoperative clinicopathologic factors and breast magnetic resonance imaging features can predict ductal carcinoma in situ with invasive components. *Eur. J. Radiol.* **85**, 780–789 (2016). 26971424 10.1016/j.ejrad.2015.12.027

[26] Lee, K. H. et al. Predictive factors for the presence of invasive components in patients diagnosed with ductal carcinoma in situ based on preoperative biopsy. *BMC Cancer* **19**, 1201 (2019). 31822268 10.1186/s12885-019-6417-3 PMC6902548

[27] Schrading, S., Schild, H., Kühr, M. & Kuhl, C. Effects of tamoxifen and aromatase inhibitors on breast tissue enhancement in dynamic contrast–enhanced breast MR imaging: a longitudinal intraindividual cohort study. *Radiology* **271**, 45–55 (2014). 24475835 10.1148/radiol.13131198

[28] Do, L.-N. et al. Predicting underestimation of invasive cancer in patients with core-needle-biopsy-diagnosed ductal carcinoma in situ using deep learning algorithms. *Tomography* **9**, 1–11 (2022). 36648988 10.3390/tomography9010001 PMC9844271

[29] Cook, E. F. & Goldman, L. Empiric comparison of multivariate analytic techniques: advantages and disadvantages of recursive partitioning analysis. *J. Chron. Dis.* **37**, 721–731 (1984). 6501544 10.1016/0021-9681(84)90041-9

