# Supplementary material for: Magnetic resonance imaging insights from active surveillance of women with ductal carcinoma in situ
Source: NPJ Breast Cancer. 2024 Aug 4;10:71. doi: 10.1038/s41523-024-00677-9 (PMC11298531; doi:10.1038/s41523-024-00677-9)

ONLINE SUPPLEMENT

Greenwood H, et al. Magnetic Resonance Imaging Insights from an Active Surveillance (AS) Sub-cohort of Women with Ductal Carcinoma in Situ.

Table of Contents

Supplementary Table 1 2
Supplementary Table 2 3
Supplementary Figure 1 3
Supplementary Table 3 4
Supplementary Table 4 5
Supplementary Figure 3 6
Supplementary Figure 2 6

Supplementary Table 1

MRI questions evaluated for patients on an Active surveillance treatment plan.

Imaging features assessed at each MRI	
Imaging Feature	***Description***
1. What is the Background Parenchymal enhancement (BPE)?	A) Minimal<br>B) Mild<br>C) Moderate<br>D) Marked
2. How distinct is the lesion from BPE?	A) No Abnormal Enhancement<br>B) Not distinct<br>C) Distinct
3. What is the likelihood of baseline invasive cancer?	A) Low<br>B) Intermediate<br>C) High
**Additional imaging features assessed at each subsequent MRI**	
Imaging Feature	***Description***
4. Has the BPE changed since the previous MRI?	A) Decrease<br>B) No Change<br>C) Increase
5. Has the lesion changed since the previous MRI?	A) Decrease<br>B) No Change<br>C) Increase
6. What is the likelihood of new or progressed DCIS?	A) Low<br>B) Intermediate<br>C) High
7. What is the likelihood of new or progressed invasive cancer?	A) Low<br>B) Intermediate<br>C) High

Supplementary Table 2

Proportionality tests comparing clinical and MRI features between patients who progressed to invasive cancer, and patients who did not have invasive cancer (patients with DCIS at surgery and patients who are still on active surveillance).

Data	P-value	Proportion	Fraction
**Premenopausal**	0.83	0.375 (IDC) / 0.446 (non-IDC)	6/16 (IDC) | 21/47 (non-IDC)
**Postmenopausal**	0.83	0.625 (IDC) / 0.553 (non-IDC)	10/16 (IDC) | 26/47 (non-IDC)
**Grade 1**	0.32	0.062 (IDC) / 0.212 (non-IDC)	1/16 (IDC) | 10/47 (non-IDC)
**Grade 2**	0.05	0.75 (IDC) / 0.425 (non-IDC)	12/16 (IDC) | 20/47 (non-IDC)
**Grade 3**	0.49	0.187 (IDC) / 0.319 (non-IDC)	3/16 (IDC) | 15/47 (non-IDC)
**Age < 50**	0.62	0.312 (IDC) / 0.425 (non-IDC)	5/16 (IDC) | 20/47 (non-IDC)
**Age ≥ 50**	0.62	0.68 (IDC) / 0.57 (non-IDC)	11/16 (IDC) | 27/47 (non-IDC)
**Minimal BPE + Distinct Lesion**	0.0003	0.5 (IDC) / 0.064 (non-IDC)	8/16 (IDC) | 3/47 (non-IDC)

Supplementary Figure 1

Cohort selection for Active Surveillance Imaging Analysis. A subset of patients from *Glencer et al.*[1] (N=72 patients, N=73 cases of DCIS) who accepted endocrine therapy was evaluated in this report. Patients who did not accept endocrine therapy were removed, and a final total of N = 62 patients (N=63 cases of DCIS) were evaluated.

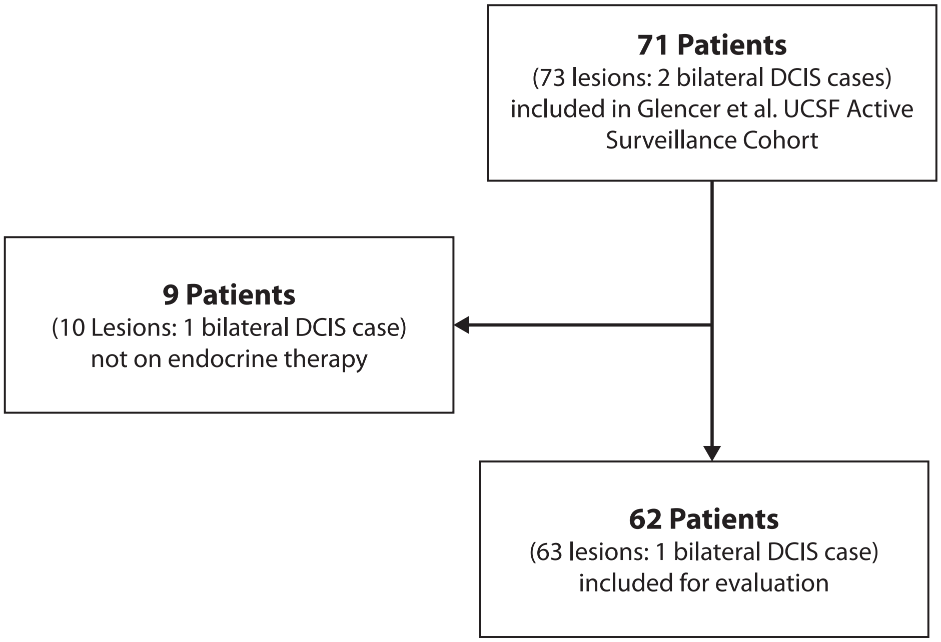

[1] Glencer, A. C. *et al.* Identifying Good Candidates for Active Surveillance of Ductal Carcinoma in Situ: Insights from a Large Neoadjuvant Endocrine Therapy Cohort. *Cancer Res Commun* **2**, 1579–1589 (2022).

Supplementary Table 3

Clinical characteristics of all patients in the cohort

Data Category	Group A (n=32)	Group B (n=17)	Group C (n=14)
**Mean Age at Diagnosis** (range in yr)	52.4 (29.8-72.6)	54.2 (41.8-77.8)	56.4 (45.5-78.8)
Age < 50	15 (46.9%)	7 (41.2%)	3 (21.4%)
Age > 50	17 (53.1%)	10 (58.8%)	11 (78.6%)
**Mean Follow-up** (range in yr)	7.9 (2.3-19.4)	8.1 (2.1-21.5)	10.5 (4.2-18.3)
**Mean Time on AS Total** (range in yr)	5.1 (0.4-18.3)	5.1 (0.2-19.3)	4.2 (0.7-9.3)
**Mean Time on AS Before Surgery** (range in yr)	1.5 (0.4-3.1)	1.6 (0.2-4.8)	3.4 (0.7-6.7)
**Mean Time on AS and No Surgery** (range in yr)	7.4 (2.9-18.3)	8.3 (2.1-19.3)	7.4 (4.2-9.3)
**Menopausal Status**			
Premenopausal	15 (46.9%)	8 (47.1%)	4 (28.6%)
Postmenopausal	17 (53.1%)	9 (52.9%)	10 (71.4%)
**HR Status**			
Positive	31 (96.9%)	15 (88.2%)	14 (100%)
Negative	0 (0.0%)	0 (0.0%)	0 (0.0%)
Unknown	1 (3.1%)	2 (11.8%)	0 (0.0%)
**HER2 Status**			
Positive	4 (12.4%)	1 (5.9%)	2 (14.3%)
Negative	14 (43.8%)	5 (29.4%)	3 (21.4%)
Unknown*	14 (43.8%)	11 (64.7%)	9 (64.3%)
**Grade**			
High	10 (31.2%)	5 (29.4%)	3 (21.4%)
Intermediate	14 (43.8%)	8 (47.1%)	10 (71.4%)
Low	7 (21.9%)	4 (23.5%)	0 (0.0%)
Unknown	1 (3.1%)	0 (0.0%)	1 (7.2%)
**Surgery**			
Yes	12 (37.5%)	8 (47.1%)	11 (78.6%)
No	20 (62.5%)	9 (52.9%)	3 (21.4%)

Supplementary Table 4

Proposed assessment for each MRI scan including change over time assessments.

**Imaging features to be assessed for semiquantitative endpoints, asked for each MRI**	
Imaging Feature	***Description***
Background Parenchymal enhancement (BPE)?	Minimal, Mild, Moderate, Marked (scale of 1-4)
Lesion type	Mass or non-mass enhancement, present or not
Lesion conspicuity/focal lesion	Lesion is very, is somewhat, is not distinct from background (scale 1-3)
**Additional imaging features assessed at each subsequent MRI (change assessment)**	
Imaging Feature	***Description***
BPE	Reduced, no change, increase (scale 1-3)
Focal lesion	More prominent, no change, less prominent (scale 1-3)

Supplementary Figure 2

Clinical interpretations after endocrine exposure. There are six different outcomes that focus on how endocrine exposure affects a lesion and/or BPE seen on MRI. Categories 1, 2, 5 and 6 are considered low risk, and 3 and 4 are considered high risk.

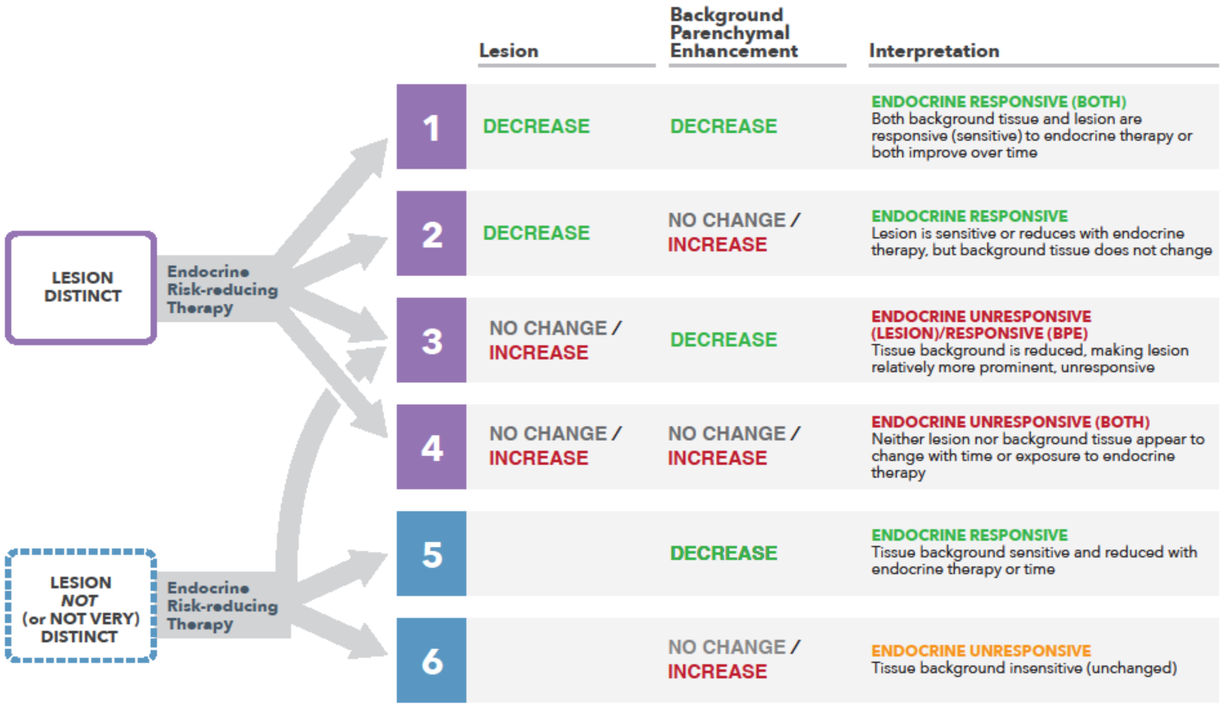

Supplementary Figure 3

Scatter plot of raw reader scores (radiologist 1 [x-axis] vs. radiologist 2 [y-axis]) for MRI questions evaluated for the first three timepoints (MR0, MR1, MR2). Percent reader agreement was assessed by total the number of answers where there was exact agreement (identical answers from both reader per patient per MR scan) divided by the total number of patients.

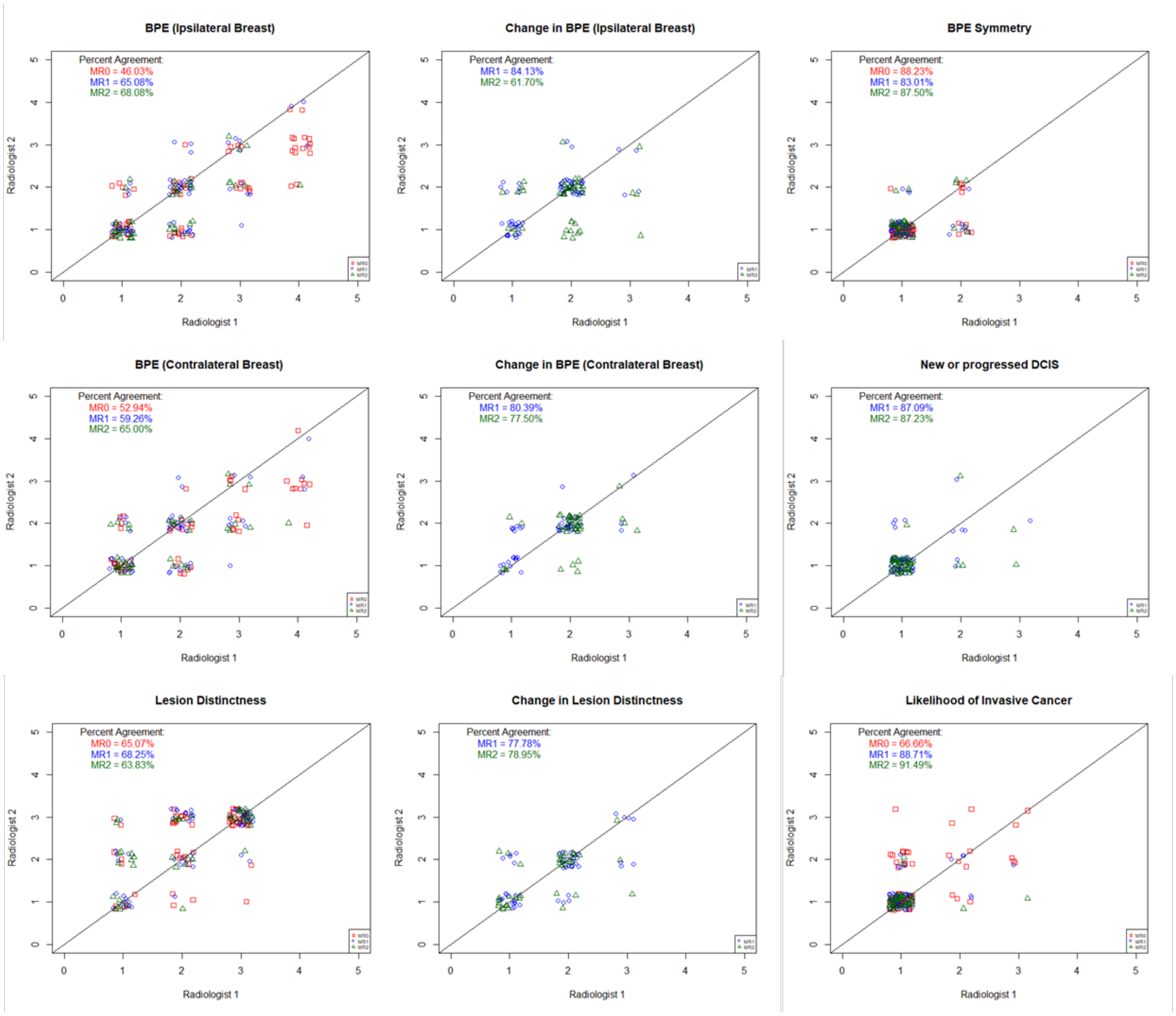

Supplement: Supplementary file 1 — Online supplement [file 41523_2024_677_MOESM1_ESM.pdf]
